# Impact of the Paralympic Games on the Beliefs of Children with Physical Disabilities Engaging in Sports and Physical Activities: A Chinese Perspective

**DOI:** 10.3390/ijerph18147296

**Published:** 2021-07-08

**Authors:** Jing Qi, Jin-He Cai, Xun Meng

**Affiliations:** College of Physical Education and Health Sciences, Zhejiang Normal University, Jinhua 321004, China; caijinhe@zjnu.edu.cn (J.-H.C.); mengxun@zjnu.edu.cn (X.M.)

**Keywords:** Paralympic Games, Beijing 2008, legacy, beliefs, physical activity

## Abstract

The purpose of this qualitative study was to explore the beliefs of Chinese children with physical disabilities engaging in sports and physical activity (PA), and the impact of the Paralympic Games on these beliefs. Five Chinese children with physical disabilities (female = 2, male = 3) were recruited for participating in the workshops of the Paralympic Games and PA, and received individual semi-structured interviews before and after the workshop implementations. Interview transcripts were analysed and presented as descriptive summaries. Three themes emerged based on the analysis of the participants’ interview data: (1) shocked, knowledgeable, and useful; (2) willingness to try, and (3) hope to obtain support. Results indicated that children with physical disabilities in this study acknowledged the positive outcomes of participating in the workshops of the Paralympic Games on the sports and PA engagement attitude change. However, children with disabilities also expressed that they need more related knowledge and information. The results of the study revealed that impairment and contextual factors (i.e., lack of support from family and physical education teachers, unsafe environments, and negative attitudes of peers without disabilities) were barriers to sports and PA engagement among children with physical disabilities in this study.

## 1. Introduction

Physical activity (PA) is defined as “any bodily movement produced by skeletal muscles that requires energy expenditure” [1]. The association of PA with health benefits in children, regardless of their disability, is well documented [2]. Despite the established health benefits of participating in PA, children with physical disabilities generally have an inactive lifestyle [3,4,5,6,7,8]. Physical inactivity and sedentary behaviours lead to deconditioning, in which children with physical disabilities showed reduced levels of cardiorespiratory fitness, thereby further decreasing PA, resulting in physical deterioration and increased difficulties in managing everyday activities [9,10]. Moreover, inactivity can contribute to social isolation and depression [11]. Hence, increasing PA levels is essential for children with physical disabilities. Increasing participation in PA requires behavioural change, as it requires more than a movement skill, as defined by the International Classification of Functioning, Disability and Health [12,13]. Factors associated with PA behaviour among children with physical disabilities, such as attitude, self-efficacy, and the number of adapted/accessible sports facilities, should ideally establish interventions aimed at increasing PA [14,15,16]. Although several studies have evaluated the effectiveness of physical training (e.g., strengthening, endurance training, and balance training) aimed at improving PA of children with physical disabilities [17,18], research conducted towards the intervention on beliefs or attitudes towards sports and PA engagement of children with physical disabilities is limited [19,20].

The Paralympic Games have grown into one of the world’s largest sporting events, with a track record for driving social inclusion, taking place every 4 years in the same year as the Olympic Games. Hosting the Olympic and Paralympic Games is a ‘once in a lifetime’ event for the members of the International Olympic Committee, designed to create a lifetime legacy that delivers increased sports and PA participation for young people [21]. To our knowledge, only one study explored the legacy of the Paralympic Games for children with disabilities. Such a study [22] examined the perceptions of the Paralympic Games of eight adolescents (11–16 years old) with physical disabilities using semi-structured interviews. The results of the study found three key themes, namely, Paralympians as role models, changing perceptions of disability, and the motivating nature of the Paralympics. More studies are needed to examine the legacy of Paralympic Games to promote social change by providing opportunities for children with disabilities to engage in sports and PA [22]. Li (2010) noted that Chinese children with physical disabilities were extremely inactive and have lower PA levels compared with those without disabilities [23]. China hosted the Beijing 2008 Olympic and Paralympic Games, and paid more attention to the latter. The Paralympic Games may affect the beliefs or motivations of children and young people with disabilities to participate in sports and PA [22].

## 2. Theoretical Framework

### 2.1. The Theory of Planned Bahviour and the Beliefs of Children with Physical Disabilities

**Figure 1:**
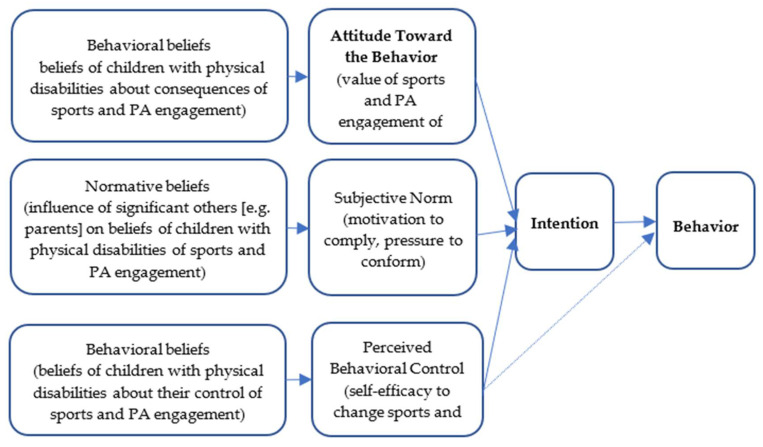
Application of the theory of planned behaviour model in sports and physical activity engagement of children with physical disabilities contexts.

Social cognitive theories have been proposed as useful ways to understand and explain health behaviours, such as tobacco use, a heart-healthy diet, and PA behaviour [24,25]. TPB is one such social cognitive theory that was used extensively within the PA domain [26,27]. TPB proposes three conceptually independent determinants of intention: behavioural beliefs, normative beliefs, and control beliefs. Behavioural beliefs refer to the beliefs about the outcome of the behaviour and evaluations of these outcomes that may produce an “attitude towards the behaviour” (AB). Normative beliefs refer to the beliefs that an individual holds based on the expectations of others (about the behaviour) including the individual’s motivation to comply with these expectations, which enables a “subjective norm” (SN). Control beliefs refer to the beliefs about any factors (e.g., skills, resources, and opportunities) that may either impede or facilitate completion of the behaviour, including the strength of each of these beliefs, and determine “perceived behavioural control” (PBC)—beliefs about the levels of personal control over the specified behaviour. AB, SN, and PBC lead to the formation of a behavioural *intention*. Ajzen indicated that if the attitude and SN are positive, then the PBC is strong, and the intention of an individual to perform the behaviour will be great [27]. The application of the TPB within sports and PA engagement of children with physical disabilities contexts is depicted in Figure 1.

From these three accessible belief systems, behavioural intention is the immediate antecedent of a particular behaviour. This study used TPB as an apposite theoretical framework. The research question that guided the study was “What is the change of the beliefs of children with physical disabilities towards engaging in PA after they participate in the workshop on the Paralympic Games and PA?” In this study, beliefs refer to the accessible beliefs [27] expressed by children with physical disabilities participating in sports and PA based on their knowledge (newly acquired and older) and previous experiences. 

### 2.2. Social Construction of Disability and Barriers to Sport and PA Engagement

Social construction theory was applied to the construct of disability to suggest the meaning ascribed by society to physical, cognitive, mental, and emotional impairments [28]. According to this theory, disability cannot exist independently of the context within which an individual interacts with the world [29]. In other words, individuals with disabilities face not only an adjustment to physical impairment or long-term illness, but also the context where they are regarded different by others. This theory has been noted as the basis of a lack of social acceptance and inclusion of people with disabilities in society [30]. The negative responses to and meanings associated with disability create the great barriers to social acceptance and inclusion [31]. 

Although evidence on the impact of PA on health-related quality of life among children with physical disabilities is rare, regular sports and PA participation has shown to have a positive association with improvements in quality of life and life satisfaction among children with physical disabilities [32,33]. Facilitators of participating in sports and PA include positive social connectedness, availability of social support, and an appropriate physical environment [34]. Unfortunately, those with physical disabilities face numerous barriers to participating in sports and PA, including impaired body functions and limited ability to perform daily tasks [35], unsupportive environments (e.g., limited local activity options, lack of access to transportation, and lack of information on accessible community-based sport and active recreational programming) [36,37,38,39,40], and lack of parental support [3]. As the TPB is closely related to the individuals’ beliefs about the presence of barriers that may prevent performance of the behaviour, examining the direct role of these specific barriers within the TPB framework could be a suitable way to address this challenge [41]. In this respect, the study uses a model of social construction of disability to capture barriers to sport and physical activities.

Based on the TPB and social construction of disability, there are two purposes in this study. The first purpose of this study was to explore the beliefs of Chinese children with physical disabilities engaging in sports and PA and the impact of the Paralympic Games on their beliefs. The second purpose of this study was to understand the barriers to sports and PA participation among children with physical disabilities.

## 3. Methods

### 3.1. Research Design

This research employed a qualitative exploratory research design using semi-structured interviews [42]. The qualitative design is described as the process of inquiry on the basis of the understanding of a social or human problem [43], and works well for research aiming to understand the perceptions of children with disabilities engaging in sports and physical activities [44,45,46].

### 3.2. Participants

Convenience sampling [47] was employed to identify candidates to participate in this study. Five children with physical disabilities in one primary school and one secondary school in Jinhua, a city in Zhejiang Province, China, participated in this study. Both the primary and secondary schools are mainstream schools. A letter describing the study’s purpose was sent to all students with physical disabilities (*N* = 11) in two schools through their physical education teachers, and five responded and expressed their willingness to participate. The participants comprised one boy in sixth grade and one girl in fifth grade in a primary school, and two boys in seventh grade and one girl in eighth grade in a secondary school. Informed consent was obtained from all participants and their parents. Pseudonyms (Xue, Ruan, Cai, Cheng, and Yuan) have been used throughout this paper to protect the privacy of the participants. Three participants in the primary school and two participants in the secondary school routinely take part in physical education lessons three or two times a week, respectively. All schools schedule 40 min per physical education lesson. All participants did not participate in physical activities specially for improving their health and movement skills. None of the participants previously participated in a program related to the Paralympic Games.

### 3.3. Program Description

This study comprised three half-day workshops, namely, the Paralympic Games and PA, covering three content areas: (a) general introduction of the Paralympic Games, (b) the introduction of the 2008 Beijing Paralympic Games, and (c) the benefits and the opportunities of sports and PA engagement for individuals with physical disabilities. Each workshop lasted approximately 1.5–2.5 h, with morning and afternoon sessions, focusing on increasing awareness of Paralympic Games and knowledge and skills involved in various physical activities. The workshops were conducted in the participants’ schools. The participants were provided with instructions in the content areas led by professionals (e.g., a lecturer teaching the history of the Olympic Games and a physical education specialist).

The first workshop is the general introduction of the Paralympic Games. This introduction comprised three parts: (a) the history of the Paralympic Games, (b) the Paralympic Games’ events and rules, and (c) the famous foreign Paralympic athletes. The lecturer introduced five foreign Paralympic athletes, three from western countries, one athlete from Africa, and one from Asia. There are American Paralympic swimmer Trischa Zorn, British Paralympic equestrian Lee Pearson, Dutch Paralympic wheelchair tennis player Esther Vergeer, South Africa’s Paralympic sprinter Oscar Pistorius, and Japanese Paralympic swimmer Mayumi Narita. After this workshop, the lecturer played videos, in which the famous Paralympic athletes won the world championship.

The second workshop is the introduction of the 2008 Beijing Paralympic Games, which include four parts: (a) the history of China’s application for hosting the 29th Olympic Games in 2008, (b) the Beijing Olympic spirits and Chinese elements of the Olympic Games (e.g., emblem, mascot, and torch) and opening and closing ceremonies, (c) China’s performance in this Paralympic Games, and (d) the introduction of the Chinese gold medal winners. Three videos were interplayed during the workshop. The first video introduced the process of bidding for the 29th Olympic Games and opening and closing ceremony segments. The second video shows the moments in which the Chinese Paralympic athletes won the world championship in the 2008 Beijing Olympic Games, such as Jianping Du winning the swimming championship of the 100 M Freestyle Men and Huaping Guo winning the judo championship of the 48 KG Women. The second set of videos include several inspirational stories of Chinese Paralympic champions, such as Jing Rong (the 2016 Rio Paralympic Games wheelchair fencing championship), Bin Hou (the 2005 Athens Paralympic Game jumping championship), and Tao Zheng (the 2016 Rio Paralympic Game 100 M backstroke championship).

The main theme of the last workshop was to introduce PA and sports for individuals with disabilities. This workshop comprised three parts: (a) the benefits of PA and PA recommendation for children from the WHO [48], (b) introduction of wheelchair sports, and (c) the opportunities to engage in various physical activities for individuals with physical disabilities.

### 3.4. Instrument

A survey of demographic data was utilised to collect basic information about the participants, such as age, gender, and grade. The authors designed an interview guide (see Table 1 and Table 2) based on the theoretical framework, previous literature, and expert opinions. Firstly, examples of possible questions related to the conceptual framework were gathered [49]. For example, in accordance with TPB, the pre-implementation interview questions were worded to capture the participants’ (a) behavioural beliefs (Questions 3 and 4 assessed the behavioural beliefs of children with physical disabilities towards PA engagement), (b) normative beliefs (Question 5 assessed the motivation of children with physical disabilities to engage in PA), and (c) control beliefs (Question 6 assessed the perceived ease or difficulty of children with physical disabilities in engaging in PA). Secondly, previous studies on perceptions of children with disabilities on sports, physical activities, or Paralympic Games were reviewed [22,50]. Thirdly, a researcher with specialisation and experience in adapted physical education offered comments and suggestions to improve the validity of the interview guides. Thereafter, a pilot study was conducted with two students with physical disabilities to identify possible problems during the interview and any confusing aspects of interview. The two students stated that they understood the interview questions completely and found no difficulty in answering the questions.

### 3.5. Procedures

The study protocol was approved by the Ethics Committee of Zhejiang Normal University (kyy2020043), and permission to conduct the study was obtained from the principals of the participating school. Informed consent forms were distributed to the participating students and their parents prior to data collection. Prior to the first interview sessions, the class teachers of each participant were contacted via email to confirm the interview arrangement. The primary researcher conducted two interviews with each participant. The first was conducted before the start of the Paralympic Games and PA workshops, and the second was conducted after the completion of the workshops. Interviews were conducted in conference rooms at the participants’ school during extracurricular periods. Interviews lasted approximately 30 min and, with permission from the interviewees and their parents, were audio recorded. During the interview sessions, key phrases, lists of major points provided by the respondents, and important quotations were documented to facilitate the post-analysis. The interview data were immediately transcribed verbatim and analysed for emerging themes of importance.

### 3.6. Data Analysis

The five interview recordings were transcribed and qualitatively analysed following the outlined procedures [49]. First, the raw data were coded for each participant based on the interview guide and included in the relevant category through deductive content analysis. Second, using inductive content analysis, the researcher identified 21 common themes or patterns shaped by cross-case raw data analysis. Third, summaries of the raw data, first-order themes, and thematic categories for participants were combined to form a hierarchical thematic structure. When differences in opinions emerged, a consensus decision was made through discussion and reassessment. The trustworthiness of this study was established using peer debriefing [43]. The researchers enlisted a peer debriefer who is an experienced qualitative researcher, to ensure the reliability and validity of the study. During data collection and analysis, the data and the researchers’ thoughts and analyses were all shared with the peer debriefer [51]. The debriefer commented on the logical nature of the researchers’ interpretations, identified all the possible categories, and informed the researchers regarding potential bias.

## 4. Results

The thematic analysis of the interview data revealed three themes relevant to the purposes of the study: (1) shocked, knowledgeable, and useful; (2) willingness to try, and (3) hope to obtain support. These themes emerged as children with physical disabilities recounted the efficacy of the programme and the impact of the workshops on their beliefs of sports and PA engagement. Several subthemes support each theme.

### 4.1. Theme 1: Shocked, Knowledgeable and Useful

Participating in the workshops provided the children with physical disabilities opportunities to enrich their knowledge and information related to the Paralympic Games and physical activities. These experiences subsequently transformed their perceptions of the Paralympic Games, sports, and PA engagement. Two subthemes have emerged: (1) feeling shocked and moved by the Paralympians and (2) enjoying these workshops but needing more.

#### 4.1.1. Subtheme 1: Feeling Shocked and Moved by the Paralympians

All participants stated that they felt shocked by the perfect performance of the athletes and were moved by their firm and indomitable spirits. Three participants indicated that they know the Paralympic Games, but they never saw the events and the moments that the Paralympians won gold medals. Yuan said, “I knew there is an Olympic Games special for individuals with disabilities, but I did not see any events on TV. My family and physical education teachers also never talked about the Paralympic Games and these athletes.” Two participants expressed that they never knew anything about the Paralympic Games and Paralympians. Cai remarked, “I never pay attention to the Paralympic Games. I think this (the workshops of the Paralympic Games) is the first time I learned this game. I really did not notice it before”. However, all participants expressed that they were totally shocked when Paralympians tried their best to fight for their countries’ glory. They used the words “awesome”, “exciting”, "cool", and “unbelievable” to describe the Paralympians. For example:

Interviewee: What did you think after watching the videos where Paralympians won the championship?

Xue: I’m excited. It’s unbelievable they can do it. They are same with me, but they can do it. They are so great!

Interviewee: Which moment touched you the most?

Xue: I think it’s the moment that their tears shed when they saw the national flags were raised.

#### 4.1.2. Subtheme 2: Enjoying These Workshops but Need More

All participants stated that the workshops were informative and useful and enjoyed these workshops. For example, Ruan said, “I never thought that there was a large sports event related to individuals with disabilities. I knew the Paralympic Games before, but I really did not pay much attention to it”. Another participant, Cai, said, “I learned about the 2008 Beijing Paralympics Game from the workshops. I am proud to be Chinese”. The participants also spoke about useful knowledge related to engaging in sports and PA. Xue said, “Actually, I never knew sports and doing various physical activities would provide people with many benefits. I learned the right way to engage in sports and physical activities that are designed for people like me. It’s very useful”.

Although the participants indicated that they learned about the Paralympic Games and physical activities from the workshops, all participants indicated that they need more related knowledge and information. Four participants indicated that they had limited opportunities to receive knowledge or information related to the Paralympic Games, Olympic Games, and other sports events, sports programmes, and various physical activities, or how to exercise in their physical education classes in school. Cheng said that he never attended physical education classes. When the researcher asked him why he was never involved in physical education classes, he said, “I generally sit in the classroom when my classmates attended the physical education classes. My physical education classes actually exist in name only.”

### 4.2. Theme 2: Willingness to Try

Before attending the workshops, participants in this study acknowledged that they had negative attitudes towards engaging in sports and PA. Participating in the workshops related to the Paralympic Games and physical activities reconstructed their beliefs and attitudes on engaging in sports and PA. Two subthemes emerged under the theme of willingness to try. These subthemes were (1) wanted to try and (2) participation barriers.

#### 4.2.1. Subtheme 1: Wanted to Try

After finishing the workshops, all participants with physical disabilities indicated that they wanted to try to engage in sports and various physical activities if they have the opportunity. Three participants expressed that they were unaware of the benefits of sports and PA engagement. For example, Yuan said, “I did not know much about the relationship among sports, PA and health and the importance of PA engagement for people’s health, especially for individuals with disabilities. I am not aware of how to participate in some sports programmes or physical activities. After attending the workshop, I now know the importance of PA engagement, and I think I will try to participate.” Two participants indicated that they were inspired by the Paralympians and were willing to try to exercise by themselves. As Xue said, “They really inspired me. I really hope I can do it like them. Though I knew it’s a dream for me, I will try to practice when given the chance.”

#### 4.2.2. Subtheme 2: Participation Barriers

After expressing positive acknowledgement, the four participants stated their concerns regarding the barriers to engaging in sports and physical activities. The barriers include negative peer attitudes and safety concerns. Negative peer attitudes of students without disabilities towards those with physical disabilities were identified by four participants as one of the barriers to participate in sports and PA. Xue said, “I do not enjoy physical education classes. I do not like participating in some sports activities. I can feel that my classmates often laugh at my movements. They must be talking about something when I did some movements. I was unwilling to participate in their activities.” Safety was the second concern to three participants. For example, Cheng expressed his concerns regarding his safety when he practised basketball during after-school periods. He said, “I played basketball in a basketball court close to my home. Several kids came towards me and practised basketball using the same court. They practised hard and bumped into me several times. It was dangerous.”

### 4.3. Theme 3: Obtaining Support

In this study, the essence of this theme captured the beliefs of children with physical disabilities supporting the future of sports and PA engagement. Two subthemes have emerged: (1) hope my family supports me and (2) hope to know how to overcome psychological barriers.

#### 4.3.1. Subtheme 1: Hope My Family Supports Me

Four participants expressed that they hoped to receive support from their families when they plan to engage in sports and PA. Firstly, they hoped that their families can learn sports knowledge, such as PA, sports programmes, the Olympic and Paralympic Games, and frequently communicate with them. For example, Yun said “I like sports. I want to know more about sports, similar to the workshops you took us into, I enjoy it very much. However, I hope my dad and mum also like sports. I hope they can tell me some good stories like the workshops told us. I like the feeling. I trust it would be perfect for me if they can do it”.

Two participants also hoped that their families can be involved in sport or physical activities with them. They thought that the support from their families were important for their sports and PA engagement.

#### 4.3.2. Subtheme 2: Hope to Know How to Overcome Psychological Barriers

All participants mentioned that they needed instructions on how to overcome psychological barriers when engaging in sports and physical activities. They expressed that they lack the confidence to participate in sports programs and various physical activities due to their limited motor abilities and poor motor performance. They were surprised by the perfect performance of the Paralympians, but they stated that they could not reach this level. For example:

Interviewee: Do you want to take part in sports or various physical activities after watching the videos that Paralympians won the championship?

Xue: Yeah, I’m very excited…they are very good. I felt I have the impulse to take part in sports when I was watching the videos, but I soon realized that I couldn’t.

Interviewee: Why?

Xue: Because I have no abilities. I couldn’t do many movements. My classmates would laugh at me when I tried to behave like them.

Interviewee: Can you give me an example?

Xue: En, let me think… oh, one time, we attended a physical education class, we practised basketball shooting. It was my turn. The teacher let me try. I tried my best to shoot, but the result was that the ball was thrown out only one step. The whole class burst into laughter. I felt embarrassed and sad at that time.

## 5. Discussion

This study examined the experiences of five children with physical disabilities participating in a PA intervention (i.e., the workshops of the Paralympic Games and PA), as interpreted from the perspectives of children with physical disabilities. Despite the small sample size, this study can offer a valuable contribution to the field by using the TPB to explain beliefs of children with physical disabilities towards sports and PA engagement. The results of this study confirm that behaviour and behavioural intention were determined by behavioural, normative, and control beliefs [27].

Overall, children with physical disabilities in this study indicate that the workshops intervention enriched their knowledge and information related to sports and PA engagement, enhanced their awareness of the disability sports and PA benefits, and strengthened their willingness and spirits in engaging in sports and PA. This finding is consistent with previous research [22] which confirmed that the legacy from the London 2012 Paralympic Games had a positive impact on enhancing PA engagement and improving the perceptions of disabilities themselves. We also targeted a part of the existing behavioural beliefs of the participants specific to being physically active (e.g., knowing the benefits of regular exercise and PA special for individuals with disabilities), to increase sports and PA engagement. These findings are also consistent with related research [52], which specified that knowing the benefits or fun of engaging in PA had a positive impact on a desire to participate in PA programmes among children with physical disabilities. This finding is in accordance with the logic of the TPB [27], which stated that behavioural beliefs and attitudes towards sports and PA engagement influenced their behavioural intentions. According to Ajzen [53], professionals can use TPB as a framework when developing interventions. This PA intervention aims to affect the intentions of children with physical disabilities to engage in sports and PA. 

Furthermore, the participants of this study stated that they need more support from their physical education teachers and family members for their sports and PA engagement, and expressed concern about negative peer attitudes towards them. This finding is in accordance with the accessible beliefs about the SN of the TPB [27,54]. The attitudes and intentions to perform a particular behaviour, in this case, engaging in sports and PA, are also influenced by SN (perceived social pressure), which are often experienced by participants with physical disabilities [55]. According to the TPB, normative beliefs provide the foundation for the SN [55]. Moreover, control beliefs are concerned with the presence or absence of factors which can facilitate or impede the performance of a particular behaviour [56]. Participants in this study concerned safety problems and their own psychological barriers to sports and PA engagement. This finding indicated that the perceptions of contextual constraints and self-efficacy (control beliefs) has a negative influence on the motivation of children with physical disabilities to engage in sprots and PA. The participants called for adding contents related to overcoming psychological barriers in the workshops, confirming the important role of perceived behaviour control belief on improving sports and PA engagement intention.

According to the research results, the workshops of the Paralympic Games and PA had a positive outcome on sports and PA engagement attitude change in children with physical disabilities in this study. However, interviews with the participants revealed that impairment and contextual factors determined the limited sports and PA engagement. This finding can be explained by the social construction of disability. Impairment was identified as a personal factor that influences sports and PA engagement among children with physical disabilities. Impairment leads to lack of competence to perform motor skills hindered the intentions of sports and PA engagement of participants. This finding supported the social construction of disability, which stated that physical impairment cannot be ignored [31].

The contextual factors, such as lack of support from family and physical education teachers, unsafe environments, and negative attitudes of peers without disabilities formed a negative sports and PA engagement context and created a barrier rather than a facilitator to support, acceptance, and inclusion. Firstly, the participants with physical disabilities in this study did not feel encouraged by their families to participate in sports and PA, which impeded their actual engagement. The involvement of family members, specifically parental involvement, is critical in ensuring the PA participation for children with disabilities [57,58]. Secondly, lack of adequate instructional modifications from physical education teachers meant having unimportant, inappropriate, and devalued roles, which led to a lack of social acceptance and decreased activities participation [59]. Moreover, the findings of this study also indicate that safety concern was one of the factors that hindered sports and PA engagement of children with physical disabilities. A group of students with disabilities in physical education classes is fraught with risk due to their high likelihood being injured; hence, the safety concerns of children with physical disabilities is easy to understand. This finding addresses the significance of physical education teachers’ beliefs and practices in their inclusive physical education classes. The role of teachers as a facilitator of including and teaching students with disabilities in their physical education classes is vital [60]. Professional training should be provided to in-service physical education teachers who handle children with disabilities in their classes. Third, children with physical disabilities in this study were excluded, and viewed as objects of curiosity in schools, thus engendering the marginalization of students with disabilities [60].

## 6. Conclusions and Limitations

The findings supported the implementation of the workshops on the Paralympic Games and PA as a strategy for favourably influencing the intention to engage in sports and PA through changing their beliefs and attitudes of children with physical disabilities toward disability sports and PA engagement. Impairment and contextual factors (i.e., lack of support from family and physical education teachers, unsafe environments, and negative attitudes of peers without disabilities) were barriers to sports and PA engagement among children with physical disabilities in this study.

We acknowledged three limitations of this study. Firstly, the participants in this study were obtained from a convenience sample in primary and secondary schools. In this study, children with physical disabilities did not represent a random sample. Therefore, the relationship between the beliefs and attitudes of children with physical disabilities in this study and those of the general population is unknown. Secondly, the intention changes were evident after the interviews. Generalising whether these changes will have a long-term influence is difficult. Further research is needed to address actual behavioural changes. Thirdly, the design of the PA intervention of this study did not include the psychological intervention contents. Further research should address the intervention related to strengthening the self-efficacy of children with physical disabilities on their potential intention of sports and PA engagement.

## Figures and Tables

**Table 1:** Pre-implementation Interview Guide.

No	Interview Questions
1	I want to talk to you about the Paralympic Games. Can you tell me what you know about the Paralympic Games? (Follow-up: Who participates? What sports do they play in the Paralympic Games? When are the Paralympic Games? Where do you find out the information about the Paralympic Games? What do you know about the Olympic Games?)
2	What do you think about the Paralympic Games?
3	What do you think about sports and PA engagement? (Follow-up: Do you participate in sports or engage in various physical activities? What sports do you participate in?)
4	What do you think about sports and PA for people with disabilities, especially for people with physical disabilities?
5	Do your families, friends or teachers encourage you to take part in sports or various physical activities? To what degree are you motivated to comply with how others feel about your PA engagement? Why?
6	How confident are you in taking part in sports or engaging in various physical activities? Why? What factors facilitate your sports participation or PA engagement? What factors impede your sports participation or PA engagement?

Note: PA: physical activity.

**Table 2:** Post-implementation Interview Guide.

No	Interview Questions
1	Describe your overall impressions of the workshops of the Paralympic Games and PA? Did it meet your expectations? Did you learn something from the workshops?
2	Describe your overall impressions of the Paralympic Games and the 2008 Beijing Paralympic Games?
3	Were the workshops effective at introducing students with disabilities to the knowledge about the Paralympic Games and PA engagement?
4	Think about the Paralympic Games. Do you think they encourage children with disabilities to take part in sports or various physical activities? Why?
5	Has the Paralympic Games changed how you think about the sport and various physical activities your take part in? Did the Paralympic Games encourage you to engage in more sports and PA? Why?
6	Would you adopt the knowledge and skills you learned from the workshops when you take part in sports or engage in various physical activities?

Note: PA: physical activity.

## Data Availability

The data presented in this study are available on requestion from the corresponding author. The data are not publicly available in compliance with the investigation confidential.
